# Cyclothymic‐hypersensitive temperament in early adolescence: Longitudinal measurement invariance and associations with psychopathology over time

**DOI:** 10.1002/jcv2.70084

**Published:** 2025-12-08

**Authors:** Anna Pezzella, Pietro Buono, Nadia Marani, Luisa Almerico, Alessandra Carta, Maria Nobile, Carmela Bravaccio, Gennaro Catone, Vincenzo Paolo Senese, Simone Pisano

**Affiliations:** ^1^ Department of Psychology Campania University ‘Luigi Vanvitelli’ Caserta Italy; ^2^ Directorate General of Health Naples Italy; ^3^ Department of Translational Medical Sciences Federico II University Naples Italy; ^4^ Department of Medicine, Surgery and Pharmacy Child and Adolescent Neuropsychiatry Unit University Hospital of Sassari Sassari Italy; ^5^ Child Psychopathology Unit Scientific Institute IRCCS Eugenio Medea Bosisio Parini Lecco Italy; ^6^ Department of Educational Psychological and Communication Sciences ‘Suor Orsola Benincasa’ University Naples Italy

**Keywords:** cyclothymic‐hypersensitive temperament, early adolescent psychopathology, longitudinal associations, longitudinal measurement invariance

## Abstract

**Background:**

Cyclothymic‐Hypersensitive Temperament (CHT) is characterised by mood instability, interpersonal hypersensitivity, and emotional hyperreactivity, traits frequently observed in general population as well as clinical settings but often eluding strict diagnostic classifications. CHT offers valuable insights into adolescents' emotional and behavioural patterns, but further research is needed to explore its longitudinal associations with psychopathology and clarify its clinical significance.

**Methods:**

Data were collected from 780 students (50.1% male, mean age at first assessment: 11.35 ± 0.49 years). The study used a longitudinal design with two assessments 2 years apart. Psychopathological symptoms were assessed using the Strength and Difficulties Questionnaire, whereas CHT Questionnaire (CHTQ) assessed CHT. Longitudinal Measurement Invariance of the CHTQ was tested to ensure construct consistency, and a Cross‐Lagged Panel Model (CLPM) was used to examine bidirectional relationships between CHT and psychopathology over time.

**Results:**

The CHTQ demonstrated configural, metric, scalar, and strict invariance throughout time, confirming its robustness and stability. The CLPM revealed a substantial stability of CHT across time (*b* = 0.430, SE = 0.078, 95% CI [0.277, 0.583], *β* = 0.475, *p* < 0.001) and a specific significant longitudinal association between CHT and psychopathology (*b* = 0.290, SE = 0.103, 95% CI [0.088, 0.492], *β* = 0.256, *p* < 0.001).

**Conclusion:**

The demonstrated invariance underscores the CHTQ's value as a reliable tool for future research on developmental trajectories and longitudinal patterns of CHT in early adolescence. CHT plays a key role in the onset of early psychopathological symptoms, positioning it within a psychopathological framework.

## Key Points

**What is known?**

- Cyclothymic‐Hypersensitive Temperament (CHT) describes a temperamental pattern characterised by emotional reactivity, impulsivity, interpersonal sensitivity, and subthreshold mood fluctuations.
- In adolescents, CHT has been associated with both internalising and externalising difficulties, yet its developmental course remains underexplored.

**What is new?**

- This study establishes the Longitudinal Measurement Invariance of the Cyclothymic‐Hypersensitive Temperament Questionnaire over a 2‐year period in early adolescence.
- Results show that CHT is stable across time and prospectively associated with higher levels of emotional and behavioural symptoms.

**What is relevant?**

- Evaluating CHT in early adolescence may support early recognition of youths at higher risk for developing mood and behavioural symptoms.
- Integrating CHT screening into clinical and school‐based settings could help inform targeted preventive interventions before full clinical onset.

## INTRODUCTION

The nature of the relationship between temperament and psychopathology remains a subject of ongoing discussion. Specifically, it is unclear whether temperament serves as a genetically driven predisposing factor for psychopathology or represents the ‘normal’ end of a spectrum, with psychopathology occupying the impairing extreme (Frick, 2004). This contrast, often framed as the vulnerability model versus the spectrum model, holds significant implications for both research and clinical practice. Investigating the pathways linking specific temperamental traits to particular forms of psychopathology may offer a means to integrate these models (Nigg, 2006) and facilitate the development of innovative measurement tools for studying these relationships (Lahey, 2004). A direct examination of the potential developmental trajectories connecting temperament and psychopathology requires a longitudinal design, with temperament assessed as a key variable.

Affective temperament types—namely depressive, hyperthymic, cyclothymic, irritable, and anxious—have been shown to serve as potential precursors to mood disorders, influencing symptom presentation, specific clinical phenotypes, and treatment outcomes (Guerreiro et al., 2013; Rihmer et al., 2010). Among these, the cyclothymic temperament (CT) is characterised by habitual, subsyndromal biphasic mood shifts, often abrupt, manifesting through subjective experiences and behavioural patterns (Perugi & Akiskal, 2002). It is associated with a high degree of emotional and behavioural instability as well as over‐reactivity (Perugi & Akiskal, 2002). Research indicates that these mood shifts are particularly prominent during interepisodic and premorbid phases of mood disorders (Akiskal & Akiskal, 1996; Brancati et al., 2021; Eaton et al., 2023; Kochman et al., 2005). When these mood fluctuations become more intense, they give rise to the construct of cyclothymia, considered an exaggerated form of CT (Parker et al., 2012; Perugi et al., 2017). Cyclothymia is marked not only by frequent and rapid mood shifts but also by heightened emotional reactivity, irritability, anxiety, poor impulse control, and increased interpersonal sensitivity (Parker et al., 2012; Perugi et al., 2017). These traits often present as exaggerated responses to perceived judgement or rejection, such as peer rejection, and contribute significantly to relational instability (Parker et al., 2012). To better capture the emotional and behavioural dimensions associated with mood instability, interpersonal hypersensitivity, and emotional hyperreactivity—traits that frequently elude strict diagnostic categories but are commonly observed in clinical practice—researchers have proposed an expanded phenotype referred to as Cyclothymic‐Hypersensitive Temperament (CHT; Hantouche et al., 2001; Kochman et al., 2005; Pisano et al., 2020).

In adulthood, a substantial body of research has demonstrated strong associations between CT and various psychopathological outcomes. Specifically, it has been shown to predict bipolar disorders, broader bipolar spectrum disorders, as well as major depressive episodes (Di Florio et al., 2010; Karam et al., 2023; Li et al., 2023; Mauer et al., 2020; Mendlowicz et al., 2005; Savitz et al., 2008; Yin et al., 2022). Additionally, it is closely linked to impaired psychosocial functioning and the presence of borderline personality traits, further underscoring its significance as a risk factor for both fully developed mood disorders and subthreshold mood symptoms (DeGeorge et al., 2014; Mendlowicz et al., 2005; for an extensive review of temperament literature in adult population, see also Favaretto et al., 2024).

On the other hand, during adolescence research has focussed mainly on the broader construct of CHT, yet available studies remain limited. Kochman et al. (2005) found that CHT in depressed children and adolescents significantly predicts later development of bipolar disorder, particularly a subtype characterised by rapid mood shifts, conduct issues, and suicidality. Similarly, Masi et al. (2018, 2023) demonstrated cross‐sectional and longitudinal associations between CHT and non‐suicidal self‐injuries as well as suicidality in adolescents. Pisano et al. (2020) identified two correlated dimensions within CHT in early adolescents: moodiness/hypersensitivity, which is more strongly associated with internalising symptoms, and impulsiveness/emotional dysregulation, which is more closely linked to externalising symptoms. A subsequent study further validated these findings in clinical populations, showing that CHT traits are associated with impaired functioning and can help differentiate clinical patients from healthy peers, with impulsiveness/emotional dysregulation notably higher in bipolar patients (Pisano et al., 2022).

While these studies underscore the importance of CHT as a significant and valid construct for understanding complex emotional and behavioural patterns in youth—going beyond the categorical DSM‐5 diagnosis—there is broad consensus that both its exact nature and clinical relevance remain to be fully clarified (Masi et al., 2023; Parker et al., 2012; Pisano et al., 2022). CHT symptoms can fluctuate over time, with certain manifestations emerging at specific developmental stages, such as adolescence, while others may appear later. This temporal variability, combined with the uncertain clinical significance of these patterns, highlights the necessity of longitudinal research to better understand the predictive and incremental validity of this construct. In this regard, the limited studies examining the stability of CT (Karam et al., 2023; Kawamura et al., 2010) suggest a substantial degree of stability over time (Placidi et al., 1998). However, these findings are constrained by methodological limitations, including the use of heterogeneous approaches, small sample sizes, and a lack of investigation into external correlates.

Based on these considerations, the present study utilised the CHT Questionnaire to examine the stability of CHT over time and its longitudinal association with general psychopathology in a preadolescent population. The CHT Questionnaire (CHTQ; Hantouche et al., 2001; Kochman et al., 2005; Pisano et al., 2020, 2022) was selected because it has been proven to be a valid measure of CHT, showing adequate reliability, validity, and measurement invariance across gender, age groups, and clinical and non‐clinical populations (Pisano et al., 2020, 2022). However, its longitudinal invariance had not yet been assessed. This aspect is crucial, as longitudinal invariance of measures is a prerequisite for longitudinal studies to ensure that repeated measurements are validly and meaningfully comparable over time (Bashkov & Finney, 2013). Consequently, to achieve the objectives of the present study, the longitudinal invariance of the CHTQ was preliminarily verified, and then the relationship between CHT and general psychopathology was investigated using a Cross‐Lagged Panel Model (CLPM) (Orth et al., 2021). This model was chosen because it allows to study the direction and dynamics of relationships between variables, that is, CHT and psychopathological symptoms, over time. Additionally, it accounts for each variable's stability and reciprocal influences, offering valuable insights into complex, time‐dependent processes.

We formulated four hypotheses. First, we hypothesised that the CHTQ would demonstrate longitudinal measurement invariance(LMI), ensuring that it consistently measures the same underlying construct over time in early adolescents, thus providing a solid foundation for further analysis. Second, based on previous population studies (Karam et al., 2023; Kawamura et al., 2010; Placidi et al., 1998), we expected that CHT would remain stable over time. Third, drawing from previous cross‐sectional studies (Karam et al., 2023; Pisano et al., 2020, 2022; Yin et al., 2022), we hypothesised a relevant association between CHT and psychopathology at the same time points. Finally, based on prior longitudinal studies (DeGeorge et al., 2014; Kochman et al., 2005; Wichstrøm et al., 2018), we expected that CHT would be longitudinally associated with psychopathological symptoms, over and above the stability of each variable over time (autoregressive effects).

## METHODS

### Participants and procedures

This study is part of a larger school survey, the Bullying and Youth Mental Health Naples Study (BYMHNS), investigating the relationship between personality traits, mental health, and common social issues such as bullying, cyberbullying, and mobile phone use (see Catone et al., 2019, 2020; Muratori et al., 2021; Pisano et al., 2019, 2020). The data presented and analysed here are being published for the first time. The study focuses specifically on the longitudinal component of the project, specifically analysing a subset of the data collected after 2 years. The first administration (T1) occurred during the 2015/2016 school year with a sample (*N* = 1048) of 6th‐grade students from 12 secondary schools in Naples, Italy (six within the city and six in peripheral areas). The second administration (T2) took place 2 years later, when the same students were in 8^th^ grade. The same protocol was administered on both occasions. Data from 780 students who took part in both assessments (50.1% male, mean age at T1 = 11.35 ± 0.49 years) were included in the analyses. The research team introduced and explained the project to students, teachers and parents, clarifying that the data would be collected anonymously and used exclusively for research purposes. After obtaining consent from both students and their parents, data were collected in the presence of the researchers. Further details on procedures and recruitment are available in previous publications (Catone et al., 2019, 2020; Muratori et al., 2021; Pisano et al., 2019, 2020). The study was approved by the Ethics Committee of the University of Campania ‘Luigi Vanvitelli’.

### Measures

#### Cyclothymic‐hypersensitive temperament questionnaire

The CHTQ is a self‐report scale designed to measure CHT in children/adolescents aged 7–18 years (Hantouche et al., 2001; Kochman et al., 2005). This measure is based on Akiskal's construct of CT in adults (Akiskal & Mallya, 1987) and the Cyclothymic Subscale of the *Temperament Evaluation of Memphis*, *Pisa*, *Paris*, *and San Diego* auto questionnaire (TEMPS‐Akiskal & Akiskal, 2005). The CHTQ consists of 25 items exploring the presence of temperamental characteristics, emotional instability, and mood swings and impulsiveness (e.g., ‘I alternate between feeling low and high according to what is going on around me’; ‘I am sometimes bubbling with energy, and at other times sluggish’; ‘When watching a film, I often get overemotional I can't help crying, being scared, or laughing’; ‘When I'm irritated, I can do stupid things I wouldn't have done otherwise’). Participants respond using a binary ‘YES’ or ‘NO’ scale, corresponding to scores of 1 or 0, respectively. In this study, we used the recently validated 22‐item Italian version of the scale, which excluded three items from the original version (items 2, 8, and 17; Pisano et al., 2020, 2022). This 22‐item version identified two highly correlated factors, impulsiveness/emotional dysregulation and moodiness/hypersensitiveness, demonstrating that the scale adequately captures both the two‐factor structure and the single dimension of CHT. The Italian 22‐item CHTQ has been proven to be a valid measure, showing adequate reliability, validity, and measurement invariance across gender, age, and clinical and non‐clinical populations (Pisano et al., 2020, 2022). The CHTQ showed satisfactory internal consistency in the present sample, with Cronbach's *α* = 0.767 at T1 and *α* = 0.766 at T2.

#### Strength and difficulties questionnaire

The Strengths and Difficulties Questionnaire (SDQ) (Goodman, 1997) is a self‐report measure designed to assess general psychopathology over the past 6 months. It comprises 25 items addressing five distinct areas: emotional symptoms, conduct problems, hyperactivity/attention difficulties, peer relationship issues, and prosocial behaviour. Each item is rated on a 3‐point scale from ‘not true’ (=0) to ‘certainly true’ (=2). In this study, we used the Italian self‐report version of the SDQ for adolescents aged 11–17 years, available on the official website (https://www.sdqinfo.org/), which has demonstrated excellent psychometric properties and validity (Corvasce et al., 2022; Di Riso et al., 2010). In this study, we considered the subscales of emotional symptoms, conduct problems, hyperactivity/inattention, and peer relationship difficulties. For each subscale a total score was computed by summing all the relative items. Each subscale score ranged from 0 to 10, with higher scores indicating greater levels of psychopathological symptoms (Cronbach alphas >0.75).

### Statistical analyses

Initially, descriptive analyses were conducted to provide an overview of the CHTQ and SDQ total scores at both time points. Subsequently, analyses were carried out in two stages to test the study hypotheses. First, LMI of the CHTQ was tested to ensure that the scale consistently measured the same construct over time. Next, a CLPM was applied to examine the stability of CHT over time, assess the concurrent associations between CHT and psychopathology at each time point, and investigate the reciprocal relationships between CHTQ and SDQ scores over time and controlling for the autoregressive effects. All statistical analyses were performed using LISREL software (version 10.3.4.4).

#### Descriptive statistics

As a preliminary step, descriptive statistics were computed for the total CHTQ and SDQ scores at T1 and T2. Means, standard deviations, and observed score ranges were calculated.

#### Longitudinal measurement invariance

The LMI of the CHTQ was assessed using a stepwise approach. To ensure that the instrument consistently measured the same underlying construct across different time points, we conducted a series of hierarchical tests with different constraints, evaluating respectively configural, metric, scalar, and strict invariance respectively (Liu et al., 2017). First, we tested configural invariance (CI) to verify that the same two‐factor structure remained stable over time. Next, metric invariance (MI) was tested by constraining the factor loadings to be equal across time points, ensuring that the items measured the constructs consistently across occasions. Scalar invariance was then tested by additionally constraining the intercepts to the equality across time points, allowing us to determine whether the mean levels of the latent variables were comparable over time. Finally, strict invariance was evaluated by constraining the residual variances to be equal across time points, verifying that the measurement errors remained consistent over time. Polychoric correlations, asymptotic covariance matrices and robust maximum likelihood estimation methods (*RML*) were used to test CFA models. Moreover, to evaluate and compare the models, we used the Maximum Likelihood (*MLχ* ^*2*^) goodness‐of‐fit test statistics in combination with other fit statistics that are less sensitive to the influence of sample size and accounting for model complexity (Cheung & Rensvold, 2002; Kline, 2023): Root Mean Square Error of Approximation (*RMSEA*), Comparative Fit Index (*CFI*), and Non‐Normed Fit Index (*NNFI*). The difference in *MLχ* ^*2*^ statistics (*MLχ* ^*2*^_*diff*_), CFI values (*CFI*_*diff*_; Cheung & Rensvold, 2002) and RMSEA (*RMSEA*_*diff*_) were used to compare the relative fits of the hierarchical estimated models.

#### Cross‐lagged panel model

A CLPM was tested to investigate the reciprocal relationships between CHT and psychopathology over time. The parcel approach was used for the measurement part of the model to get a simplified yet robust representation of the latent factors (Matsunaga, 2008). For the CHTQ, a single latent factor was modelled and represented by four parcels. These parcels were created by grouping items from the two CHTQ dimensions, with two parcels derived from each dimension. Items were grouped to ensure that the parcels were balanced in terms of factor loadings. For the SDQ, a single latent psychopathology factor was modelled, represented by four parcels. Each parcel was constructed by summing the items from the four SDQ subscales: emotional symptoms, conduct problems, hyperactivity, and peer problems. The CLPM included autoregressive paths to account for the stability of each variable over time (e.g., CHT at Time 1 predicting CHT at Time 2), concurrent associations to capture the relationships between CHT and SDQ at the same time points (e.g., CHT at Time 1 correlating with SDQ at Time 1), and cross‐lagged paths to assess the predictive effects between CHT and psychopathology over time (e.g., CHT at Time 1 predicting psychopathology at Time 2, and vice versa). Model fit was evaluated using the same indices as in the measurement invariance analysis (i.e., *RMSEA*, *CFI*, and *NNFI*).

## RESULTS

### Descriptive statistics

Means, standard deviations, and observed score ranges for the total CHTQ and SDQ scores at T1 and T2 are reported in Table 1.

**TABLE 1:** Descriptive statistics for total CHTQ and SDQ scores at Time 1 and Time 2.

Measure	Time 1				Time 2			
	*M*	SD	Min	Max	*M*	SD	Min	Max
CHTQ total	9.28	4.23	0	21	10.08	4.35	0	21
SDQ total	9.97	5.99	0	38	10.31	5.58	0	29

*Note*: CHTQ = Cyclothymic‐Hypersensitive Temperament Questionnaire; *M* = mean; SD = standard deviation; SDQ = Strengths and Difficulties Questionnaire.

### Longitudinal measurement invariance

In the first step, we tested CI across the two time points (Model A). The results indicated a good model fit, with *RMSEA* = 0.094, *CFI* = 0.972, *NNFI* = 0.970, and *SB‐MLχ* ^*2*^ (874, *N* = 780) = 1250.62, *p* < 0.001 (see Table 2). This suggests that the two‐factor structure of the model remained consistent across the two time points. In the second step, we tested MI (Model B). The model showed a good fit, with *RMSEA* = 0.093, *CFI* = 0.971, *NNFI* = 0.969, and *SB‐MLχ* ^*2*^ (896, *N* = 780) = 1287.32, *p* < 0.001. The more constrained model did not show a significant reduction in fit compared to the previous model, *MLχ* ^*2*^_*diff*_ (22) = 36.7, *p* = 0.026, *CFI*_*diff*_ = −0.001, *RMSEA*_*diff*_ = −0.001, indicating that factor loadings were equivalent across time points, and items contributed similarly to the latent variables. In the third step, we tested scalar invariance (Model C). The results again indicated a good fit, with *RMSEA* = 0.093, *CFI* = 0.966, *NNFI* = 0.965, and *SB‐MLχ* ^*2*^ (916, *N* = 780) = 1377.346, *p* < 0.001. The comparison with the previous model revealed no significant reduction in fit, *MLχ* ^*2*^_*diff*_ (20) = 90.026, *p* < 0.001, *CFI*_*diff*_ = −0.005, *RMSEA*_*diff*_ = 0. This suggests that item intercepts remained stable, allowing meaningful comparisons of latent means across time points. Finally, strict invariance was tested (Model D). The model also showed a good fit, *RMSEA* = 0.092, *CFI* = 0.965, *NNFI* = 0.965, and *SB‐MLχ* ^*2*^ (938, *N* = 780) = 1410.228, *p* < 0.001. Also in this case, the comparison with the scalar model revealed no significant reduction in fit, *MLχ* ^*2*^_*diff*_ (22) = 32.88, *p* = 0.064, *CFI*_*diff*_ = −0.001, *RMSEA*_*dif*f_ = −0.001, confirming that the residual variances were stable across both time points. In summary, these results provide strong evidence that the CHTQ demonstrates a full measurement longitudinal invariance, supporting its validity as a robust and stable tool for longitudinal research.

**TABLE 2:** Longitudinal measurement invariance analysis across T1 and T2: Multi‐group hierarchical confirmatory factor analyses goodness‐of‐fit indices as a function of the study (*N* = 780).

Model	*RMSEA*	*CFI*	*NNFI*	*SB‐MLχ* ^2^	*df*	*MLχ* ^2^_*diff*_	*df*_*diff*_	*CFI*_*diff*_	*RMSEA*_*diff*_
Model A	0.094	0.972	0.970	1250.62***	874	–	–	–	–
Model B	0.093	0.971	0.969	1287.32***	896	36.70*	22^a^	−0.001	−0.001
Model C	0.093	0.966	0.965	1377.35***	916	90.01***	20^b^	−0.005	0
Model D	0.092	0.965	0.965	1410.23***	938	32.88	22^c^	−0.001	−0.001

*Note*: Model A: two‐factor configural invariance (CI). Model B: two‐factor CI and metric invariance (MI). Model C: two‐factor CI, MI, and scalar invariance (SI). Model D: two‐factor CI, MI, SI, and invariant uniquenesses (IU).

^a^ The reference model is Model A.

^b^ The reference model is Model B.

^c^ The reference model is Model C.

***p < .001; **p < .01; *p < .05.

### Cross‐lagged panel model

**FIGURE 1:**
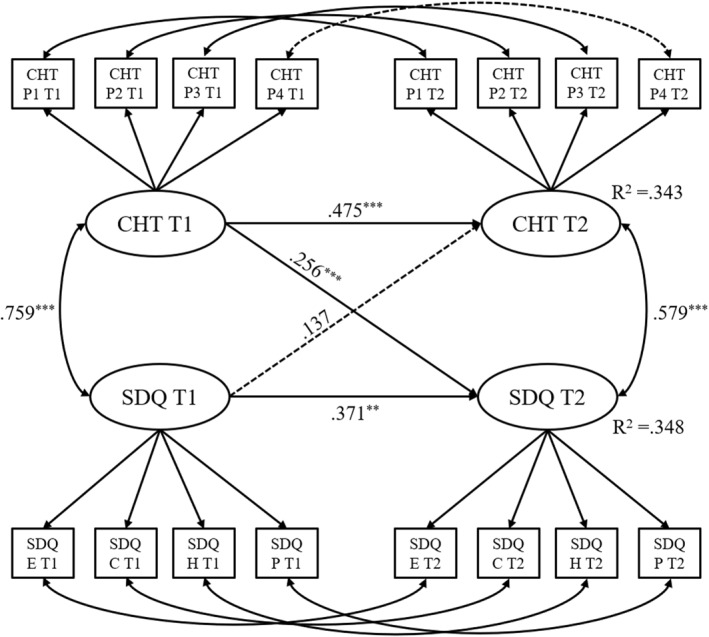
Cross‐lagged Panel Model examining the bidirectional relationships between cyclothymic‐hypersensitive temperament and psychopathological symptoms across two time points (**p < .01; ***p < .001).

The model showed acceptable fit indices, with *RMSEA* = 0.073, 90% CI [0.067, 0.079], *NNFI* = 0.919, and *CFI* = 0.930. As shown in Figure 1, the results revealed significant autoregressive effects for both constructs. Higher CHT scores at T1 were positively associated with higher CHT scores at T2, *b* = 0.430, SE = 0.078, 95% CI [0.277, 0.583], *β* = 0.475, *p* < 0.001, and higher SDQ scores at T1 were positively associated with higher SDQ scores at T2 *b* = 0.300, SE = 0.077, 95% CI [0.149, 0.451], *β* = 0.371, *p* < 0.001, indicating considerable stability in both CHT and psychopathological symptoms over time. Additionally, the associations between CHT and SDQ were significant and strong at each time point, with *r* = 0.759, *p* < 0.001 at T1 and *r* = 0.579, *p* < 0.001 at T2, respectively. In terms of cross‐lagged associations, data indicated that CHT at T1 was significantly associated with SDQ at T2, *b* = 0.290, SE = 0.103, 95% CI [0.088, 0.492], *β* = 0.256, *p* < 0.001. This suggests that higher levels of CHT at the first time point (T1) were associated with higher levels of psychopathological symptoms at T2 beyond the stability of SDQ over time. In contrast, SDQ at T1 was not significantly associated with CHT at T2, *b* = 0.089, SE = 0.055, 95% CI [‐0.019, 0.197], *β* = 0.137, *p* = 0.056, beyond the stability of CHT over time. Notably, the model remained consistent, even when controlling for gender differences in scores at T2, underscoring the robustness of the findings. Finally, the *R* ^*2*^ values indicate that the model explained 34.3% of the variance in CHT at T2 and 34.8% of the variance in SDQ at T2. These values represent the proportion of variance in each construct at T2 accounted for by the autoregressive and cross‐lagged effects. Overall, the results highlight a strong association between the constructs at the same time points and that CHT is linked to higher subsequent levels of psychopathological symptoms.

## DISCUSSION

The objectives of this study were to assess the measurement integrity and longitudinal stability of the CHTQ in an early adolescent sample and to explore reciprocal associations between CHT and psychopathological symptoms over time.

The results from LMI confirm that the CHTQ demonstrates configural, metric, scalar, and strict invariance, supporting its reliability in measuring the same construct across time. This evidence extends previous validations of the CHTQ's invariance across groups (Pisano et al., 2020, 2022) by establishing its longitudinal invariance. The two‐factor structure of the tool—consisting of two highly correlated factors previously named impulsiveness/emotional dysregulation and moodiness/hypersensitivity—has been confirmed, supporting the multidimensional nature of CHT, reflecting both internalising and externalising features (Pisano et al., 2020, 2022). Notably, this study further confirmed the robust psychometric property of the CHTQ, marking a significant advance in the validation of this instrument. Such confirmation is crucial for ensuring that developmental changes observed in longitudinal studies reflect genuine variations in CHT rather than inconsistencies in measurement. This robust invariance highlights the CHTQ's utility as a reliable instrument for future research on developmental trajectories and longitudinal patterns of CHT in adolescent populations, enabling accurate and valid comparisons over time.

The results from the CLPM provide valuable insights into the stability and associations of CHT over time, underscoring its significance in understanding adolescent psychopathology. First, the stability of the CHT construct, as demonstrated by significant autoregressive effects, aligns with previous research on temperament, which consistently highlights the enduring nature of cyclothymic traits (Karam et al., 2023; Kawamura et al., 2010; Placidi et al., 1998). Although this stability is expected due to the inherent consistency of temperament traits, these findings are particularly notable as they stem from a sample of early adolescents—a developmental stage characterised by rapid emotional and psychological changes (Tijssen et al., 2010). Similarly, the stability of SDQ scores over time corroborates previous findings in both clinical and non‐clinical samples, further reinforcing the reliability of these measures in tracking emotional and behavioural symptoms during early adolescence (Blok et al., 2022; Isdahl‐Troye et al., 2022). Beyond the stability, the study reveals significant positive associations between CHT and psychopathological symptoms, both concurrently and longitudinally. Specifically, CHT scores at both time points were positively correlated with SDQ‐measured psychopathological symptoms, consistent with previous cross‐sectional studies (Karam et al., 2023; Pisano et al., 2020, 2022; Yin et al., 2022). Additionally, higher CHTQ scores at T1 were linked to elevated psychopathological symptoms at T2, highlighting that CHT may be a key factor in emotional and behavioural difficulties over time. These associations remained robust even after controlling for gender, further validating the generalisability of the findings. The observed longitudinal link between CHT and psychopathology holds significant theoretical importance, as CHT remains an inadequately defined construct, with ongoing challenges in clarifying its clinical characteristics. Our findings build on previous research by demonstrating that CHT plays a crucial role in the development of early psychopathological symptoms, reinforcing the pivotal role of temperament in the onset of psychopathology (Akiskal & Akiskal, 1996; Karam et al., 2023; Kochman et al., 2005; Li et al., 2023; Mauer et al., 2020; Syrstad et al., 2020). While these findings derive from a general population sample, they highlight pathways that may be even more pronounced in clinical populations; however, direct clinical extrapolation should be cautious until replicated in clinical cohorts. Although this study focuses on a non‐clinical sample, it offers a more direct perspective by positioning CHT within a psychopathological framework, rather than viewing it merely as a normative temperamental trait. These findings align with the predisposition explanation in the existing literature, which suggests that extreme manifestations of certain temperamental traits may predispose young people to the emergence of specific psychopathological symptoms, acting as significant risk factors for psychiatric disorders (Wichstrøm et al., 2018). This relationship is particularly salient during early adolescence, a developmental stage characterised by emotional instability and the onset of mood and behavioural disorders. Our findings contribute to the growing body of research demonstrating a strong association between CHT and various psychopathological conditions, including the bipolar disorders, borderline personality traits, interpersonal difficulties and adverse outcomes such as non‐suicidal self‐injury and heightened suicidal ideation (DeGeorge et al., 2014; Kochman et al., 2005; Masi et al., 2018, 2023; Perugi et al., 2017). Similarly, research on adolescents with Attention Deficit Hyperactivity Disorder and age‐matched healthy controls has shown that negative affect dimensions play a pivotal role in driving dysregulated behaviours and emotions (Carta et al., 2022). This underscores the importance of understanding temperamental profiles as key indicators for early mental health and behavioural problems, facilitating timely detection and targeted interventions.

This study has some limitations. First, the sample is limited to a general school‐based population, not allowing us to draw conclusion on clinical population. Replicating this research in clinical samples would provide a deeper understanding of the relationship between CHT and psychopathological symptoms in mood and behavioural disorders, helping to clarify the complex boundaries of this construct and identify diagnostic categories with a higher prevalence of cyclothymic traits. Second, while the CLPM is useful for analysing reciprocal associations between CHT and psychopathology, it does not establish causation or predictive effects. Moreover, the use of a traditional CLPM, which aggregates within‐persons and between‐persons variance, does not differentiate between temporary variations and stable individual differences. A Random Intercept Cross‐Lagged Panel Model (RI‐CLPM) would have been more appropriate to separate these effects (Usami et al., 2019); however, its application requires at least three time point assessment, whereas our study included only two assessment waves. Future research with additional time points could adopt the RI‐CLPM to more accurately examine the associations and reciprocal effects between CHT and psychopathology. Third, in our CLPM we modelled both CHT and psychopathology as single latent constructs, as the present work represented an initial and foundational step aimed at exploring the longitudinal association between CHT and overall psychopathological vulnerability. Nevertheless, this approach entailed a loss of specificity, as it precluded the examination of dimension‐level associations and finer nuances within both CHT and psychopathology. Future studies with larger samples, additional assessment waves, and theoretically driven models should therefore investigate dimension‐specific cross‐lagged associations to clarify whether the different CHT dimensions show unique developmental trajectories and distinct longitudinal links with specific domains and nuances of psychopathology. Fourth, as data were collected from schools in a single Italian region, cultural and contextual factors may have influenced the expression of CHT, and replication in other cultural settings will be important to support generalisability. Finally, reliance on self‐report measures may introduce response bias; future studies could enhance data validity by incorporating multi‐informant assessments; in this regard a parent version of CHTQ would be a valuable addition.

## CONCLUSION

Despite its limitations, this study addresses a critical gap in research on CHT during adolescence. Importantly, it establishes a foundation for the CHTQ as a reliable and stable tool for assessing CHT across time. Our findings reinforce the hypothesised link between CHT and psychopathological manifestations in early adolescence, highlighting the potential of integrating the CHTQ into screening contexts to facilitate the early identification of vulnerable individuals and enable timely, targeted interventions. These results carry significant theoretical and practical implications, supporting psychopathology models that identify difficult temperament or self‐regulation deficits as early risk factors for psychiatric disorders (Beauchaine & McNulty, 2013; Bridgett et al., 2015). While this study underscores the potential of CHT as an early vulnerability marker, further research on clinical populations is needed to refine our understanding of the relationship between CHT and psychopathology. Such investigations could contribute to enhancing diagnostic criteria and developing more effective intervention strategies for at‐risk individuals.

## AUTHOR CONTRIBUTIONS

**Anna Pezzella**: Data curation; formal analysis. **Pietro Buono**: Writing—original draft; supervision. **Nadia Marani**: Data curation**. Luisa Almerico**: Data curation; writing—original draft. **Alessandra Carta**: Writing—original draft; supervision. **Maria Nobile**: Methodology; writing—original draft. **Carmela Bravaccio**: Methodology; writing—review and editing; supervision. **Gennaro Catone**: Conceptualization; investigation; writing—review and editing. **Vincenzo Paolo Senese**: Conceptualization; data curation; methodology; formal analysis. **Simone Pisano**: Conceptualization; methodology; writing—review and editing; supervision.

## CONFLICT OF INTEREST STATEMENT

The authors declare no conflicts of interest.

## ETHICAL CONSIDERATIONS

The Ethics Committee of the University of Campania ‘Luigi Vanvitelli’ approved the study protocol (No. 500 of 29/04/2016). Informed consent was obtained from the parents of all participants involved in the study.

## Data Availability

The data that support the findings of this study are available from the corresponding author upon reasonable request.
